# Supplementary material for: Over-Expressing TaSPA-B Reduces Prolamin and Starch Accumulation in Wheat (Triticum aestivum L.) Grains
Source: Int J Mol Sci. 2020 May 5;21(9):3257. doi: 10.3390/ijms21093257 (PMC7247331; doi:10.3390/ijms21093257)
Supplement: Supplementary file 1 [file ijms-21-03257-s001.zip › ijms-766224-supplementary/Figure S1.docx]

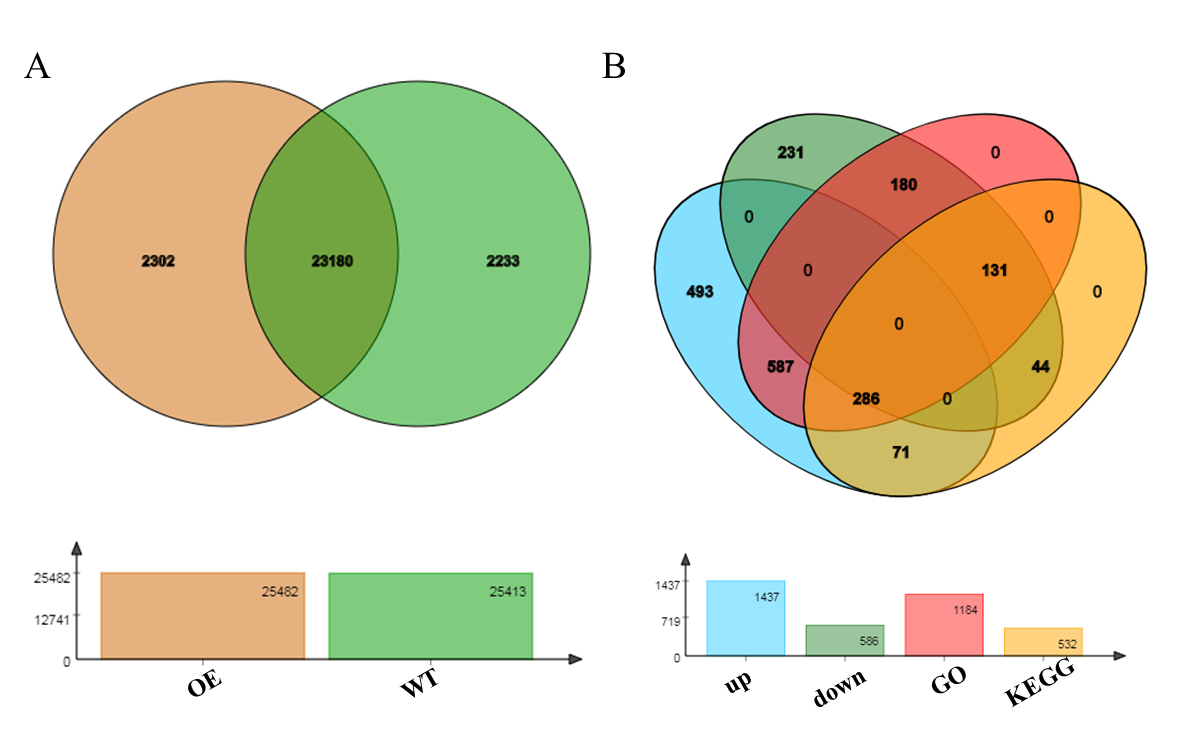


**Figure** **S1.** Gene annotation and DEG analysis. (A) Venn diagram of genes annotated to the reference genome of CS. (B) Venn diagram of DEGs enriched in GO terms and KEGG pathways. DEGs are identified with a threshold of |log_2_FC|>1 and FDR < 0.05.
