# Supplementary material for: Over-Expressing TaSPA-B Reduces Prolamin and Starch Accumulation in Wheat (Triticum aestivum L.) Grains
Source: Int J Mol Sci. 2020 May 5;21(9):3257. doi: 10.3390/ijms21093257 (PMC7247331; doi:10.3390/ijms21093257)
Supplement: Supplementary file 1 [file ijms-21-03257-s001.zip › ijms-766224-supplementary/Table S5.docx]

# **Table S5.** Statistics of differentially expressed transcription factor genes

| TF family | Upregulated genes | Downregulated genes |
| --- | --- | --- |
| AP2 | 1 | 2 |
| ARF/B3 |  | 1 |
| B3 | 2 | 4 |
| bHLH | 6 | 1 |
| bZIP | 15 | 3 |
| C2H2 |  | 1 |
| CPP |  | 2 |
| DBB | 2 |  |
| Dof |  | 4 |
| E2F/DP |  | 2 |
| ERF | 6 |  |
| GRAS | 4 |  |
| HB-other | 2 | 4 |
| HD-ZIP/HB-other |  | 3^1^ |
| LBD (AS2/LOB) | 1 | 3 |
| MIKC/M-type | 2 |  |
| MYB | 3 | 9 |
| MYB_related | 7 | 7 |
| NAC | 15 | 15 |
| RAV/ERF/B3 | 1 |  |
| WRKY | 6 |  |

Note: ^1^The three genes contain HD-ZIP and HB-other domains.
