# Supplementary material for: Over-Expressing TaSPA-B Reduces Prolamin and Starch Accumulation in Wheat (Triticum aestivum L.) Grains
Source: Int J Mol Sci. 2020 May 5;21(9):3257. doi: 10.3390/ijms21093257 (PMC7247331; doi:10.3390/ijms21093257)
Supplement: Supplementary file 1 [file ijms-21-03257-s001.zip › ijms-766224-supplementary/Table S6.docx]

# **Table S6.** Primers used for qRT-PCR analysis

| Fragment name | Forward primer (5′-3′) | Reverse primer (5′-3′) |
| --- | --- | --- |
| *GAPDH* | ttcaacatcattccaagcagc | cgtaacccaaaatgcccttg |
| *SPA^1^* | tgcgactcttaatagtgagacc | tatggaaggggaggcacaaaca |
| *SPA-A* | gatcatcacccgcaaatga | aacaagccgagaaaccactata |
| *SPA-B* | ccaagcaatgtctagcatatcatc | ggttgcatatgtagcgaaggt |
| *SPA-D* | tccgcaacgggaatctacta | aatgtccacttaacaagccaaact |
| *Bx14* | ggtgccgccccatcac | gcaggtattccccaaaatatcat |
| *By15* | ccacaaaatagagatcaattcacta | cacgagggtgatgactactgt |
| *Dx2* | agcggttagtcctctttgtgg | cggagctgctggtccatg |
| *Dy12* | gttagcgcagagcagcaag | ccctccatccgacacactg |
| *LMW-GS^2^* | TGCAGCCACACCAGATAGCTCAG | TCAGTAGGCACCAACTCCGGTAC |
| *α-gli^2^* | agacctttctcatccttgcc | tgtaccaatggaacttgctct |
| *ω-1,2 gli^2^* | tggccaatgaaatgaacacc | ggtcggggttacacattatgg |
| *ω-5 gli^2^* | caagaaccttccccatacca | caacgatgattcacccgtct |
| *r-gli^2^* | caacaattttctcagccccaaca | ttcttgcatgggttcacctgtt |

Note: *GAPDH* was the house-keeping gene used as internal control. ^1^The pair of primers was designed to amplify three homoeologous copies of *TaSPA* according to the conserved sequences. *^2^*The pairs of primers were desighed to amplify the whole multigene family member genes according to the conserved sequences.
