# Supplementary material for: Over-Expressing TaSPA-B Reduces Prolamin and Starch Accumulation in Wheat (Triticum aestivum L.) Grains
Source: Int J Mol Sci. 2020 May 5;21(9):3257. doi: 10.3390/ijms21093257 (PMC7247331; doi:10.3390/ijms21093257)
Supplement: Supplementary file 1 [file ijms-21-03257-s001.zip › ijms-766224-supplementary/Table S7.docx]

# **Table S7.** Primers used to detect positive transgenic plants

| Fragment name | Forward primer (5′-3′) | Reverse primer (5′-3′) |
| --- | --- | --- |
| *TaSPA-B^1^* | ggtaccatggagcccgtgttcttct | gagctcctacatcaccatgttgac |
| *pBar^2^* | cgtcaaccactacatcgagacaag | gctgaagtccagctgccagaaac |
| *pSPA-B^3^* | ccacaacaccgagcaccaca | gcaagaccggcaacaggatt |

Note: ^1^The pair of primers was designed to amplify the full length *TaSPA-B* gene. The uppercase letters represent the sequences of *TaSPA-B* genes. The lowercase letters represent the adapter sequences of pCAMBIA3300 vector. ^2^The pair of primers was designed to amplify the *Bar* gene of the vector. ^3^The pair of primers was designed to amplify partial *TaSPA-B* gene and promoter sequence of *Glu-1Dx5*.
